# Diagnosis, treatment, and notification of syphilis during pregnancy in the state of Goiás, Brazil, between 2007 and 2017

**DOI:** 10.11606/s1518-8787.2021055003122

**Published:** 2021-10-18

**Authors:** Iana Mundim de Oliveira, Rívert Paulo Braga Oliveira, Rosane Ribeiro Figueiredo Alves

**Affiliations:** I Universidade Federal de Goiás Hospital das Clínicas GoiâniaGO Brasil Universidade Federal de Goiás. Hospital das Clínicas. Goiânia, GO, Brasil; II Universidade Federal de Ouro Preto Departamento de Estatística Ouro PretoMG Brasil Universidade Federal de Ouro Preto. Departamento de Estatística. Ouro Preto, MG, Brasil; III Universidade Federal de Goiás Faculdade de Medicina Programa de Pós-Graduação em Ciências da Saúde GoiâniaGO Brasil Universidade Federal de Goiás. Faculdade de Medicina. Programa de Pós-Graduação em Ciências da Saúde. Goiânia, GO, Brasil

**Keywords:** Pregnant women, Syphilis, pharmacologic treatment, Serodiagnosis of Syphilis, trends, Disease Notification, Health care Quality, Access, and Evaluation, Maternal and Infant Health care

## Abstract

**OBJECTIVE:**

To analyze the evolution of syphilis during pregnancy notification regarding clinical classification, diagnosis and treatment in the state of Goiás, Brazil, between 2007 and 2017.

**METHODS:**

This is a time-series study, analyzing data provided by the Health Secretariat of the state of Goiás. The variables related to the diagnosis and treatment of pregnant women and their partners were analyzed, and their evolution trend during the years. Descriptive statistics and percentage calculation were used. Cochran-Armitage test with a significance level α = 0.05 was used to determine increase and decrease trends.

**RESULTS:**

During the period, 7,774 cases were notified. The highest percentage of notifications occurred in the second trimester of pregnancy (39.8%) and corresponded to primary syphilis (34.1%). The most frequent treatment prescribed was benzathine benzylpenicillin with a dosage of 7.2 million (43.8%). Between 2007 and 2017, there was an increasing trend in the notification percentage of latent (14.1% to 30.7%), secondary (5.2% to 19%), and tertiary syphilis (4.4% to 11.4%). The treatment with benzathine benzylpenicillin with a dosage of 7.2 million also increased (19.3% to 59.6%). The percentages of primary syphilis decreased (43.4% to 22.1%), as well as other treatments’ percentages.

**CONCLUSIONS:**

Latent syphilis notification of pregnant women and treatment with penicillin at the dosage of 7,200,000 IU increased. Notification forms’ data completeness also increased for the variables clinical classification and treatment, suggesting improvements in the notification process.

## INTRODUCTION

Syphilis is considered nowadays a serious public health problem due to high infection rates in many parts of the world^1^. It can be transmitted by sexual contact, by direct contact with lesions, or vertically during pregnancy, causing severe consequences for fetuses^2,3^. In pregnant women, the probability of adverse outcomes can increase up to 52%, including abortion, preterm labor, precocious and late congenital syphilis, fetal or neonatal death, and hospitalization^2,3^.

Several factors influence the outcome of vertical transmission, such as late diagnosis during pregnancy, inadequate or lack of treatment^4^. In Brazil, syphilis monitoring during pregnancy with serological test is determined in the first and third trimesters of pregnancy, during birth or abortion, risk situations, and cases of sexual violence^5^. Nevertheless, late syphilis diagnosis, during birth or curettage, or inadequate treatment are predominant^6,7^. Moreover, in most cases, syphilis diagnosis occurs while monitoring, without clinical manifestations. In such circumstances, the case is categorized and treated as latent syphilis of indeterminate duration or late^8^.

The Brazilian Ministry of Health recommends the treatment with benzathine benzylpenicillin by intramuscular injection, with an adequate dosage for the clinical case, starting until 30 days before birth. To reduce reinfection risk, the Ministry recommends testing and treating pregnant women’s sexual partner(s)^5^. A study about six Brazilian states showed adequate treatment rates lower than 70% in all regions researched, and less than 20% of the partners received simultaneous treatment^9^. In Goiás, almost half of the cases (46%) have a late maternal syphilis diagnosis in the second or third trimester of pregnancy. In this context, only 18% of the partners receive concomitant treatment^10^.

Congenital syphilis, an entirely preventable condition, results from non-treated or inadequately treated cases of syphilis during pregnancy and is an indicator of health care quality and a condition of mandatory notification in Brazil since 1986^11^. However, syphilis during pregnancy is notified in Brazil since 2005 and in Goiás since 2007^12,13^. Nevertheless, under-reporting is a persistent problem, as well as the inadequate or incomplete filling of notification forms, which directly interferes in the surveillance and control of the disease^6,4^. Considering the control of syphilis during pregnancy as essential to prevent congenital syphilis, and that notification is the tool to communicate and follow the cases, this study aims to analyze the syphilis during pregnancy notification evolution regarding clinical classification, diagnosis and treatment in the state of Goiás between 2007 and 2017.

## METHODS

This is a time-series study, analyzing the syphilis during pregnancy notifications to the *Sistema de Informação de Agravos de Notificação* – SINAN (Information System of Grievance Notification) between 2007 and 2017 in the state of Goiás.

This study is part of a larger research, entitled *“Sífilis materna em Goiás – análise de série histórica, de 2007 a 2017”* (Maternal Syphilis in Goiás - historical series analysis between 2007 and 2017). The Research Ethical Committee of the *Hospital das Clínicas da Universidade Federal de Goiás* approved the study, as well as the *Centro de Excelência em Ensino, Pesquisa e Projetos “Leide das Neves da SES-GO”* (decisions 3,060,244 and 3,070,206; amendments 3,499,285 and 3,568,091).

The *Superintendência de Atenção Integral à Saúde* (Superintendence of Integral Health Care) of the *Secretaria Estadual de Saúde de Goiás* – SES-GO (Goiás Health Secretariat) provided the researchers with the data regarding the forms in January 2019. Personal data (such as the patients’ name, mother’s name, and address) were omitted before the data were sent to the researchers. The following variables were collected and coded from the original database: birthdate/age, race/color, education, occupation, pregnancy stage at the notification date, syphilis clinical classification, report of conducting and the results of treponemal or nontreponemal tests, treatment and plan prescribed for the sexual partner and the pregnant woman and the justification in case of a non-treated partner. The analysis excluded patients residing in other states, even if they were notified in Goiás.

The data were analyzed descriptively using the software IBM SPSS version 23.0. Afterward, the percentages were measured for the variables clinical classification and treatment plan adopted by the number of cases in each category divided by total number of cases per year multiplied by one hundred (%). The increase or decrease percentage trends during the years were verified with the Cochran-Armitage test for trend using the software R at a significance level α = 0.05.

## RESULTS

Between 2007 and 2017, 7,774 syphilis during pregnancy cases were registered in Goiás. Pregnant women age varied between 12 and 49 years old, with a mean of 24.8 years old and a standard deviation of 6.5 years. Pardo (mixed ethnicity) women (55.4%), with primary (29.4%) or secondary education (24.2%), were the most affected.

Regarding clinical characteristics, less than one quarter of the cases were notified in the first trimester of pregnancy (21.8%). The second trimester presented the higher percentage (39.8%). The most frequent clinical condition was primary syphilis (34.1%), and latent syphilis comprised one quarter of the cases. Table 1 presents these data.

**Table 1:** Clinical and laboratory characteristics of pregnant women diagnosed with syphilis in Goiás between 2007 and 2017.

Gestational age^a^	n	% valid
First trimester	1,692	21.8
Second trimester	3,094	39.8
Third trimester	2,595	33.4

Clinical classification of Syphilis^b^	n	% valid
Primary	2,385	34.1
Secondary	1,260	18.0
Tertiary	602	8.6
Latent	1,771	25.3

Nontreponemal test (VDRL)^c^	n	% valid
Reagent	6,164	79.6
Non-Reagent	779	10.0
Unperformed	444	5.7

Treponemal test^d^	n	% valid
Reagent	6,576	84.6
Non-Reagent	183	2.4
Unperformed	593	7.6

^a^ 393 forms filled as “ignored”.

^b^ 776 notifications without information, and 980 filled as “ignored”.

^c^ 3 notifications without information, and 384 filled as “ignored”.

^d^ 3 notifications without information, and 419 filled as “ignored”.

**Figure 1:**
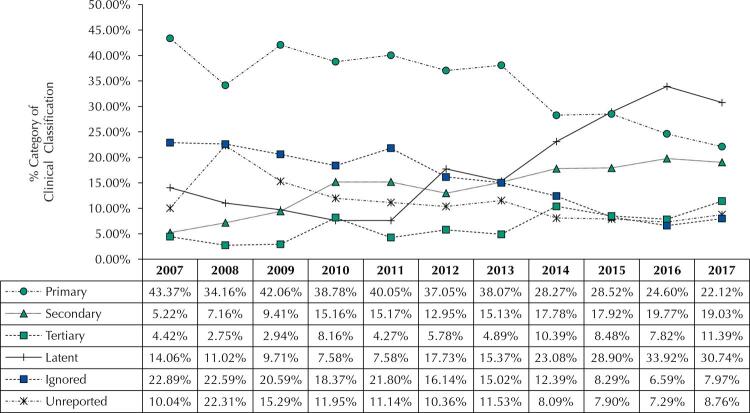
Notification percentage of maternal syphilis, according to the clinical classification in Goiás, between 2007 and 2017.

Primary syphilis diagnosis reduced 21% between 2007 and 2017, while latent syphilis increased 16%. The number of notification forms filled as “ignored” or not filled (unreported) also reduced 14% and 1.3%, as presented in Figure 1.

The most frequent treatment prescribed for pregnant women was benzathine benzylpenicillin with a dosage of 7,200,000 international units (IU) (43.8%). 7.2% of the pregnant women diagnosed were not treated, and 4.2% received other treatment (Table 2).

**Table 2:** Treatment prescribed for pregnant women and their partners in Goiás between 2007 and 2017.

Pregnant woman notified^a^	n	% valid
Benzathine penicillin G 2,400,000 U	2,503	32.2
Benzathine penicillin G 4,800,000 U	556	7.2
Benzathine penicillin G 7,200,000	3,407	43.8
Another plan	324	4.2
Unperformed	557	7.2

Partner^b^	n	% valid
Benzathine penicillin G 2,400,000 U	1,051	17.4
Benzathine penicillin G 4,800,000 U	262	4.3
Benzathine penicillin G 7,200,000	1,378	22.8
Another plan	160	2.6
Unperformed	1,893	31.3

^a^ 3 notifications without information, and 424 filled as “ignored”.

^b^ 1,729 notifications without information, and 1301 filled as “ignored”.

**Figure 2:**
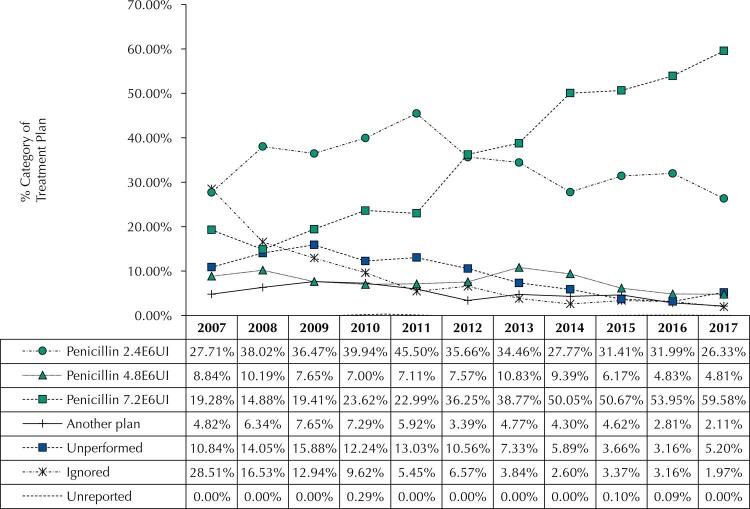
Notification percentage of maternal syphilis according to the treatment plan prescribed in Goiás between 2007 and 2017.

Figure 2 shows a 40% increase in the frequency of penicillin treatment at the dosage of 7.2 million IU in the period studied. It was also perceived a decreasing trend in the percentages of data ignored (from 28.5% in 2007 to 2.0% in 2017).

Cochrane-Armitage test confirmed the percentage increase of latent, secondary, and tertiary syphilis, and of the use benzathine benzylpenicillin at the dosage of 7.2 million IU. It presented a decreasing trend for primary syphilis, and ignored or unreported data (Table 3).

**Table 3:** Percentage trend analysis’s results for the variables clinical classification and treatment.

Clinical classification		Treatment	
	
Classification	p	Classification	p
Primary^(a1,d)^	2.2x10^-16^	Penicillin 2.4E6UI^(a1,d)^	7.6x10^-10^*
Secondary^(a2,c)^	2.2x10^-16^*	Penicillin 4.8E6UI^(a1,d)^	1.4x10^-6^*
Tertiary^(a2,c)^	4.4x10^-14^*	Penicillin 7.2E6UI^(a2,c)^	2.2x10^-16^*
Latent^(a2,c)^	2.2x10^-16^*	Another plan^(a1,d)^	1.7x10^-9^*
Ignored^(a1,d)^	2.2x10^-16^*	Ignored^(a1,d)^	2.2x10^-16^*
Unreported^(a1,d)^	1.3x10^-13^*	Unperformed^(a1,d)^	2.2x10^-16^*
		Unreported^(a3,nt)^	1.0

Alternative hypotheses: a1 – *H*_1_: *p*_2007_ > ... > *p*_2017_; a2 – *H*_1_: *p*_2007_ < ... < *p*_2017_; a3 – *H*_1_: no trend.

Conclusion: ^c^ Increasing; ^d^ decreasing; ^nt^ no trend.

* Statistically significant (Cochran-Armitage test significance level α = 0,05).

The partners were treated in 42% of the cases, considering the whole period. During the years, partner’s treatment proportion increased from 0,8% to 40%. Nevertheless, the percentage of partners notified as non-treated also increased from 1.6% to 37%. The percentage of unreported data decreased from 94% to 2% between 2007 and 2017.

The main justifications for not treating the partner informed in the notification forms were the lack of further contact with the pregnant women (24.8%), non-reagent serology (15%), and the partner’s nonattendance at the health unit (11.4%). Other justifications for the lack of partner’s treatment corresponded to 48.8% and were described subjectively, impeding their grouping. Nevertheless, each of these justifications represented a percentage lower than 0.1%.

## DISCUSSION

This study identified high proportions of inadequate diagnosis and treatment for syphilis during pregnancy in Goiás. On the other hand, a significant evolution occurred since 2007, evidenced by the higher consonance between the recent data and the current scientific evidence. In the researchers’ knowledge, these results are innovative for the study of syphilis during pregnancy in Brazil. They bring relevant contributions to evaluate state control strategies for syphilis during pregnancy and congenital syphilis and to detect flaws in the infected women’s care.

The high percentage of primary syphilis in this study (34.1%) suggests possible failures in classification because it is expected of latent syphilis with ignored or late duration to be more frequent in this population. Such findings corroborate other studies in different Brazilian cities and regions, such as in the state of Tocantins, in the north, and in the south of Brazil^7,14^. Nevertheless, our data differ from other studies, such as those presented by Souza, Rodrigues & Gomes^15^ and Marques et al.^16^, where the higher percentages were of tertiary and ignored syphilis, respectively. On the other hand, the gradual increase of latent syphilis notification indicates a possible approximation between the clinical practice and the available evidence.

This article shows a high proportion of pregnant women with syphilis treated inadequately. In more than 10% of the cases, no therapeutic plan was adopted or a plan without penicillin. Scientists have long known that penicillin is the only medication that prevents vertical transmission of syphilis, as well as a safe, efficient, and low cost alternative^17^. It was impossible to quantify the exact percentage of inadequate treatments in this study. Nevertheless, the fact that one third of the sample was treated with a dosage of 2,400,000 IU signals a high percentage. High rates of inadequate or no treatment were also found in other localities in Brazil and the world^18^.

In 2015, a national shortage of penicillin was announced, and the medication reservation for pregnant women with syphilis was recommended due to the absence of other efficient options^21^. Nevertheless, the period with higher use of alternate treatments to penicillin in this study was 2009–2010. Therefore, the shortage seems to be unrelated to the increase in inadequate treatment.

Still about the variable treatment, the results of the stratified analysis demonstrate a gradual reduction of the use of benzathine benzylpenicillin at the dosage of 2,400,000 IU and a yearly increase of the dosage of 7,200,000 IU. This fact matches the increase of latent syphilis notifications. The adequate identification of the clinical phase and treatment of syphilis during pregnancy is fundamental to reach the global goal of reducing congenital syphilis, as well as the recommended strategies to strengthen prenatal care and increase the testing^22^.

The frequency of partner(s) treated in this study (42.9%), described in Table 2, although below the ideal, is higher than what is presented in other national and international studies^9,23^. The high level of under-reporting is highlighted, especially in the first years of the series. However, with the successive increase of all filling categories, the unreported data decreased during the years. Although studies show the increase of partners treated, the data about them are still neglected^25^. Regarding the justifications for partner non-treatment, other studies also presented the absence of further contacts with the partner and reagent serology as the major reasons^7^. It is worth mentioning that sexual partners treatment is fundamental to prevent reinfection and the spread of new cases. Therefore, the monitoring of infected individuals’ partners is reasonable, even if they did not have sexual intercourse after the woman diagnosis. In this context, testing sexual partners is important to define the therapeutic plan. Nevertheless, the current notification form omits such data, which impedes the analysis of the real situation of partners’ care. We emphasize the responsibility of health services in summoning partners to visit the health unit, offering the laboratory test timely.

Finally, an important finding is the apparent increase in notification forms filling in the variables clinical classification and treatment, although still lower than the ideal. Although the notification is an important step to control the disease in the state, the quality of the record is still low, presenting under-reporting and incomplete filling, among other problems^26^. In this context, the complete filling of the notifications might be impeded by the lack of data at the notification moment, and the presence of the options “ignored” and “not applicable” in the forms, even for mandatory or essential data. The last institutional form was presented in 2008, in which it was reviewed for the last time. Thus, more studies about the completeness of the notifications are recommended, as well as the updating of the form and its filling recommendations.

This study presents limitations inherent to use of secondary data, such as the possibility of insufficient registers, fails and incompleteness in the notification forms. The State Health Secretariat provided the data directly, which increases their quality.

Considering the results, the updating and implementation of institutional protocols aligned with scientifical evidence is necessary. In this context, prenatal care staff training and the notification of syphilis cases during pregnancy are of the utmost importance. Such actions are essential to reach the global goal of reducing congenital syphilis.

## CONCLUSIONS

This study findings demonstrate high proportions of inadequate diagnosis and treatment of syphilis during pregnancy. Nevertheless, notification of latent syphilis during pregnancy increased, as well as the treatment with benzathine benzylpenicillin at the dosage of 7,200,000 IU, which suggests an increase in the adherence to the recommended practices. The increase of data completeness in the notification forms for the variables clinical classification and treatment suggests an enhancement of the notification process during the years.
