# Supplementary material for: Diagnosis, treatment, and notification of syphilis during pregnancy in the state of Goiás, Brazil, between 2007 and 2017
Source: Rev Saude Publica. 2021 Oct 18;55:68. doi: 10.11606/s1518-8787.2021055003122 (PMC8522754; doi:10.11606/s1518-8787.2021055003122)
Supplement: Translation

# Translation: Diagnóstico, tratamento e notificação da sífilis durante a gestação em Goiás, de 2007 a 2017

**Authors:** Iana Mundim de Oliveira, Rívert Paulo Braga Oliveira, Rosane Ribeiro Figueiredo Alves

**Affiliations:** I Brasil Universidade Federal de Goiás. Hospital das Clínicas. Goiânia, GO, Brasil; II Brasil Universidade Federal de Ouro Preto. Departamento de Estatística. Ouro Preto, MG, Brasil; III Brasil Universidade Federal de Goiás. Faculdade de Medicina. Programa de Pós-Graduação em Ciências da Saúde. Goiânia, GO, Brasil

**Keywords:** Gestantes, Sífilis, tratamento farmacológico, Sorodiagnóstico da Sífilis, tendências, Notificação de Doenças, Qualidade, Acesso e Avaliação da Assistência à Saúde, Serviços de Saúde Materno-Infantil

## Abstract

**OBJETIVO:**

Analisar a evolução das notificações da sífilis durante a gestação em relação à classificação clínica, ao diagnóstico e ao tratamento no estado de Goiás, entre 2007 e 2017.

**MÉTODOS:**

Estudo de série temporal com análise de dados fornecidos pela Secretaria Estadual de Saúde de Goiás. Foram analisadas as variáveis relacionadas ao diagnóstico e tratamento das gestantes e seus parceiros, e sua tendência evolutiva ao longo dos anos. Utilizou-se estatística descritiva, cálculo de porcentagens e verificação das tendências de aumento e diminuição por meio do teste de Cochran-Armitage com nível de significância α = 0,05.

**RESULTADOS:**

Ao todo, 7.774 casos foram notificados no período. A maior porcentagem das notificações ocorreu no segundo trimestre de gestação (39,8%) e correspondeu à sífilis primária (34,1%). O tratamento prescrito com maior frequência foi a penicilina benzatina em dose de 7,2 milhões (43,8%). Entre 2007 e 2017, observou-se tendência crescente nas porcentagens de notificações de sífilis latente (14,1% para 30,7%), secundária (5,2% para 19%) e terciária (4,4% para 11,4%), assim como no tratamento com penicilina benzatina em dose de 7,2 milhões (19,3% para 59,6%). Tendência decrescente foi observada nas porcentagens de notificação de sífilis primária (43,4% para 22,1%) e nos demais esquemas de tratamento.

**CONCLUSÕES:**

Houve aumento no número de notificações de sífilis latente em gestantes e no tratamento com penicilina na dose de 7.200.000 UI. Também foi observado aumento na completitude dos dados da ficha de notificação nas variáveis de classificação clínica e tratamento, sugerindo melhora no processo de notificação.

## INTRODUÇÃO

A sífilis é atualmente considerada um grave problema de saúde pública devido às elevadas taxas de prevalência em várias partes do mundo^1^. Pode ser transmitida tanto sexualmente, através de contato direto com lesões, quanto verticalmente durante a gestação, ocasionando consequências graves para o feto^2,3^. Estima-se que, em gestantes infectadas, a probabilidade de ocorrer desfechos adversos pode aumentar em até 52%, isto inclui abortamento, parto pré-termo, sífilis congênita precoce e tardia, óbito fetal ou neonatal e aumento do número de internações hospitalares^2,3^.

Diversos fatores influenciam no desfecho da transmissão vertical, como diagnóstico tardio na gestante, tratamento inadequado ou não realizado^4^. No Brasil, o rastreamento para sífilis durante a gestação por meio de testes sorológicos é preconizado no primeiro e no terceiro trimestres de gestação, no momento do parto ou aborto, ou em situações de risco ou violência sexual consumada^5^. Todavia, observa-se na prática o predomínio do diagnóstico tardio da sífilis, no momento do parto ou curetagem, e de tratamento inadequado em gestantes^6,7^. Observa-se ainda, que na maioria dos casos, o diagnóstico de sífilis é feito durante o rastreio, portanto, na ausência de qualquer manifestação clínica. Nestes casos, recomenda-se classificar e tratar como sífilis latente de duração indeterminada ou tardia^8^.

O Ministério da Saúde considera como tratamento adequado aquele realizado com penicilina benzatina por via intramuscular, em dose adequada para a forma clínica, e iniciado em até 30 dias antes do parto. Para reduzir o risco de reinfecção, também é recomendada a testagem e o tratamento para o(s) parceiro(s) sexual(is) da gestante^5^. Um estudo realizado em seis unidades federativas brasileiras demonstrou taxas de tratamento adequado inferiores a 70% em todas as regiões pesquisadas, sendo que menos de 20% dos parceiros receberam tratamento simultaneamente^9^. Em Goiás, quase metade dos casos (46%) tem o diagnóstico de sífilis materna realizado tardiamente, no segundo ou terceiro trimestre de gestação. Nesse contexto, apenas 18% dos parceiros recebem tratamento concomitante^10^.

A sífilis congênita, um agravo totalmente prevenível, que resulta da sífilis gestacional não tratada ou tratada inadequadamente, comporta-se como indicador da qualidade da assistência e é um evento de notificação obrigatória desde 1986^11^. Já a sífilis durante a gestação é notificada no Brasil desde 2005 e, em Goiás, desde 2007^12,13^. No entanto, a subnotificação é um problema persistente, assim como o preenchimento inadequado ou incompleto da ficha de notificação, o que interfere diretamente na vigilância e controle da doença^6,4^. Considerando que o controle da sífilis durante a gestação é um importante fator para prevenção da sífilis congênita, e que a notificação é o meio de comunicação e acompanhamento dos casos, este estudo teve como objetivo analisar a evolução das notificações da sífilis durante a gestação em relação à classificação clínica, ao diagnóstico e ao tratamento no estado de Goiás, entre 2007 e 2017.

## MÉTODOS

Trata-se de um estudo de série temporal, com análise das notificações de sífilis durante a gestação ao SINAN, realizadas entre 2007 e 2017, no estado de Goiás.

Este estudo integra um maior, intitulado “Sífilis materna em Goiás – análise de série histórica, de 2007 a 2017” e foi aprovado pelo Comitê de Ética em Pesquisa do Hospital das Clínicas da Universidade Federal de Goiás e pelo Centro de Excelência em Ensino, Pesquisa e Projetos “Leide das Neves da SES-GO” (pareceres 3.060.244 e 3.070.206 e emendas 3.499.285 e 3.568.091).

As informações provenientes das fichas foram fornecidas aos pesquisadores pela Superintendência de Atenção Integral à Saúde da Secretaria Estadual de Saúde de Goiás (SES-GO) em janeiro de 2019. Dados pessoais ou de identificação dos sujeitos, como nome do paciente, nome da mãe e endereço foram omitidos previamente ao envio do banco aos pesquisadores. Do banco original, foram coletadas e posteriormente codificadas as seguintes variáveis: data de nascimento/idade, raça/cor, escolaridade, ocupação, idade gestacional na data da notificação, classificação clínica da sífilis, a informação de realização e o resultado de testes não treponêmicos e treponêmicos, de tratamento e esquema prescrito ao parceiro sexual e à gestante e, em casos dos parceiros não tratados, o motivo para o não tratamento. Foram excluídos da análise os casos residentes em outros estados, mesmo tendo sido notificados em Goiás.

Os dados extraídos foram analisados de forma descritiva por meio do software IBM SPSS versão 23.0. Em seguida, as porcentagens foram calculadas para as variáveis de classificação clínica e esquema de tratamento adotado, pelo número de casos em cada categoria dividido pelo número total de casos ao ano, vezes cem (%). As tendências de aumento ou redução de porcentagens ao longo dos anos foram verificadas através do teste de tendência de Cochran-Armitage, no software R, utilizando-se nível de significância α = 0,05.

## RESULTADOS

Entre os anos de 2007 e 2017 foram registrados 7.774 casos de sífilis durante a gestação em Goiás. A idade das gestantes variou entre 12 e 49 anos, com média de 24,8 anos e desvio padrão de 6,5 anos. Foram observadas maiores proporções de mulheres pardas (55,4%), com nível primário de escolaridade (29,4%) ou ensino fundamental (24,2%).

Em relação às características clínicas, menos de um quarto dos casos foram notificados no primeiro trimestre de gestação (21,8%), sendo o maior percentual encontrado no segundo trimestre (39,8%). A forma clínica mais frequente foi a sífilis primária (34,1%) e um quarto dos casos foi classificado como sífilis latente. Os dados estão descritos na Tabela 1.

**Tabela 1:** Características clínicas e laboratoriais de gestantes diagnosticadas com sífilis em Goiás, entre 2007 e 2017.

Período gestacional^a^	n	% válida
Primeiro trimestre	1.692	21,8
Segundo trimestre	3.094	39,8
Terceiro trimestre	2.595	33,4

Classificação clínica da Sífilis^b^	n	% válida
Primária	2.385	34,1
Secundária	1.260	18,0
Terciária	602	8,6
Latente	1.771	25,3

Teste não treponêmico (VDRL)^c^	n	% válida
Reagente	6.164	79,6
Não Reagente	779	10,0
Não realizado	444	5,7

Teste treponêmico^d^	n	% válida
Reagente	6.576	84,6
Não Reagente	183	2,4
Não realizado	593	7,6

^a^ 393 preenchidas como “ignorado”.

^b^ 776 notificações sem informação e 980 preenchidas como “ignorado”.

^c^ 3 notificações sem informação e 384 preenchidas como “ignorado”.

^d^ 3 notificações sem informação e 419 preenchidas como “ignorado”.

A proporção de diagnóstico de sífilis primária reduziu 21% entre 2007 e 2017, enquanto a sífilis latente apresentou crescimento de 16%. O número de notificações preenchidas como “ignorado” ou não preenchidas (sem informação) também reduziram 14% e 1,3% respectivamente, conforme pode ser visualizado na Figura 1.

**Figura 1:** Porcentagens de notificações da sífilis materna, segundo a classificação clínica em Goiás, entre 2007 e 2017.

O tratamento prescrito com maior frequência para as gestantes foi a penicilina benzatina em dose de 7.200.000 unidades internacionais (UI) (43,8%). Em 7,2% dos casos as gestantes não foram tratadas e 4,2% das gestantes receberam outro tipo de tratamento (Tabela 2).

**Tabela 2:** Tratamento instituído para gestantes e seus parceiros em Goiás, entre 2007 e 2017.

Gestante notificada^a^	n	% válida
Penicilina G Benzatina 2.400.000 U	2.503	32,2
Penicilina G Benzatina 4.800.000 U	556	7,2
Penicilina G Benzatina 7.200.000	3.407	43,8
Outro esquema	324	4,2
Não realizado	557	7,2

Parceiro^b^	n	% válida
Penicilina G Benzatina 2.400.000 U	1.051	17,4
Penicilina G Benzatina 4.800.000 U	262	4,3
Penicilina G Benzatina 7.200.000	1.378	22,8
Outro esquema	160	2,6
Não realizado	1.893	31,3

^a^ 3 notificações sem informação e 424 preenchidas como “ignorado”.

^b^ 1.729 notificações sem informação e 1301 preenchidas como “ignorado”.

Conforme pode ser visualizado na Figura 2, no período estudado observou-se aumento de 40% na frequência de tratamento com penicilina em dose de 7,2 milhões de UI. Também foi observada tendência decrescente nos percentuais de dados ignorados (de 28,5% em 2007 para 2,0% em 2017).

**Figura 2:** Porcentagens de notificações da sífilis materna, segundo esquemas de tratamento instituídos em Goiás, entre 2007 e 2017.

O teste de Cochrane-Armitage confirmou o aumento dos percentuais de sífilis nas formas latente, secundária e terciária, e do uso de penicilina benzatina em dose de 7,2 milhões de UI. Apresentaram tendência decrescente para a forma clínica primária, bem como os dados ignorados ou sem informação (Tabela 3).

**Tabela 3:** Resultado da análise de tendência de porcentagens para variáveis classificação clínica e tratamento.

Classificação clínica		Tratamento	
	
Classificação	p	Classificação	p
Primária ^(a1,d)^	2,2x10^-16^	Penicilina 2.4E6UI ^(a1,d)^	7,6x10^-10^*
Secundária ^(a2,c)^	2,2x10^-16^*	Penicilina 4.8E6UI ^(a1,d)^	1,4x10^-6^*
Terciária ^(a2,c)^	4,4x10^-14^*	Penicilina 7.2E6UI ^(a2,c)^	2,2x10^-16^*
Latente ^(a2,c)^	2,2x10^-16^*	Outro esquema ^(a1,d)^	1,7x10^-9^*
Ignorada ^(a1,d)^	2,2x10^-16^*	Ignorado ^(a1,d)^	2,2x10^-16^*
Sem informação ^(a1,d)^	1,3x10^-13^*	Não realizado ^(a1,d)^	2,2x10^-16^*
		Sem informação ^(a3, st)^	1,0

Hipóteses alternativas: a1 – *H*_1_: *p*_2007_ > ... > *p*_2017_; a2 – *H*_1_: *p*_2007_ < ... < *p*_2017_; a3 – *H*_1_: sem tendência

Conclusão: ^c^ Crescente; ^d^ decrescente; ^st^ sem tendência.

* Estatisticamente significativo (teste de Cochran-Armitage nível de significância α = 0,05).

Em 42% dos casos os parceiros foram tratados, considerando-se o período total. Ao longo dos anos foi observado incremento das proporções de tratamento em parceiros de 0,8% para 40%, no entanto, a porcentagem de parceiros notificados como não tratados também cresceu de 1,6% para 37% dos casos. A porcentagem de dados sem informação caiu de 94% para 2% entre 2007 e 2017.

Os principais motivos para não tratamento do parceiro, informados pela ficha de notificação foram a falta de contato posterior com a gestante (24,8%), sorologia não reagente (15%) e não comparecimento do parceiro à unidade de saúde para buscar tratamento (11,4%). Os outros motivos para não tratamento do parceiro, que correspondem a 48,8%, foram descritos de maneira subjetiva impossibilitando o agrupamento, no entanto, apresentaram porcentagens menores que 0,1%, individualmente.

## DISCUSSÃO

Este estudo identificou elevadas proporções de diagnóstico e tratamento inadequados para sífilis durante a gestação, em Goiás. Por outro lado, demonstrou uma evolução satisfatória nestes aspectos desde 2007, evidenciada pela maior consonância entre os dados mais recentes e as evidências científicas atuais. Até onde se verificou, estes são resultados inovadores para o estudo da sífilis durante a gestação no Brasil. Os resultados deste estudo trazem contribuições relevantes na avaliação das estratégias de controle da sífilis durante a gestação e da sífilis congênita implementadas no estado, bem como na detecção de pontos passíveis de melhoria quanto ao manejo das gestantes infectadas.

A elevada porcentagem de sífilis primária neste estudo (34,1%) sugere uma possível falha na classificação, quando se espera que o diagnóstico de sífilis latente com duração ignorada ou tardia seja mais frequente nesta população. Estes achados corroboram aqueles descritos em outras cidades e regiões brasileiras, como Tocantins na região norte, e na região sul do Brasil^7,14^. No entanto, estes dados diferem de outros estudos como o de Souza, Rodrigues e Gomes^15^e o de Marques et al.^16^, em que os maiores percentuais foram de sífilis terciária e ignorada, respectivamente. Por outro lado, o aumento gradual de notificações de sífilis latente aponta para uma possível aproximação entre a prática clínica e as evidências disponíveis.

Este artigo revela uma proporção elevada de gestantes com sífilis tratadas inadequadamente. Em mais de 10% dos casos notificados não foi adotado nenhum esquema terapêutico ou foi adotado algum esquema diferente da penicilina. Há muito se sabe que a penicilina é a única droga capaz de prevenir a transmissão vertical da sífilis, além de ser considerada uma alternativa segura, eficaz e de baixo custo^17^. Não foi possível quantificar a porcentagem exata de tratamentos inadequados neste estudo, no entanto, o fato de um terço da amostra ter sido tratada com dose de 2.400.000 UI sinaliza um percentual elevado. Altas taxas de tratamento inadequado ou não realizado também foram encontradas em outras localidades no Brasil e no mundo^18^.

Em 2015 foi anunciado o desabastecimento nacional de penicilina, com recomendações de reserva do medicamento para tratamento de gestantes com sífilis, devido à ausência de opções terapêuticas eficazes^21^. Entretanto, destaca-se que o período de maior utilização de esquemas alternativos que não a penicilina nesta amostra foram os anos de 2009 e 2010, portanto, este desabastecimento não parece ter contribuído para o aumento de tratamento inadequado.

Ainda sobre a variável tratamento, os resultados da análise estratificada demonstram redução gradual do uso de penicilina benzatina em dose de 2.400.000 e aumento ano a ano em dose de 7.200.000 UI, fato condizente com o aumento das notificações de sífilis latente. Ressalta-se que a identificação correta da fase clínica e a instituição do tratamento adequado para a sífilis durante a gestação são imprescindíveis para o alcance da meta global da redução da sífilis congênita, somada às estratégias de fortalecimento do pré-natal e aumento da testagem preconizados^22^.

A frequência de tratamento dos parceiros(as) sexuais neste estudo (42,9%), descrita na Tabela 2, embora aquém do desejável, mostrou-se superior em relação a outros estudos nacionais e internacionais^9,23^. Ressalta-se o alto índice de sub-registro dessa variável, sobretudo nos primeiros anos da série, porém, com aumento sucessivo de todas as categorias de preenchimento, e diminuição dos dados sem informação com o passar dos anos. Embora estudos demonstrem o aumento do número de parceiros tratados, os dados sobre eles são ainda bastante negligenciados^25^. Quanto aos motivos alegados para não tratamento do parceiro, outros estudos corroboram com este, apontando a ausência de contato posterior do mesmo com a gestante e ausência de sorologia reagente como principais motivos^7^. Convém ressaltar que o tratamento dos parceiros sexuais é um importante aliado na prevenção de reinfecção e geração de novos casos, portanto é razoável que os parceiros de pessoas infectadas sejam rastreados para sífilis, mesmo sem contato sexual posterior ao diagnóstico da mulher. Neste contexto, a realização de testes diagnósticos pelos parceiros sexuais é importante para definição do esquema terapêutico, no entanto, não é um dado presente na ficha de notificação atualmente, o que dificulta a análise da real situação de assistência aos parceiros. Ressalta-se a responsabilidade dos serviços de saúde em convocar os parceiros para comparecer à unidade de saúde e oferecer os testes laboratoriais em tempo hábil.

Por fim, um achado interessante a ser discutido é o aparente aumento da completitude da ficha de notificação observado nas variáveis de classificação clínica e tratamento, apesar de ainda estar aquém do esperado. Sabe-se que, embora o início da notificação tenha sido um grande passo para o controle da doença no estado, a qualidade do registro ainda é débil, tendo como problemas a subnotificação e o preenchimento incompleto, entre outros^26^. Nesse contexto, acredita-se que a completitude pode ser dificultada tanto pela existência de dados que inacessíveis no momento da notificação, quanto pela presença das opções “ignorado” e “não se aplica”, sobretudo nos dados considerados obrigatórios ou essenciais. Ressalta-se que o último formulário instrucional para preenchimento é do ano de 2008, data em que a ficha foi revisada pela última vez. Assim, estudos mais amplos sobre a completitude dos dados são desejáveis, bem como a atualização da ficha e das recomendações de preenchimento.

O presente estudo apresenta limitações inerentes à utilização de dados secundários, como a possibilidade de registros insuficientes, falhas durante o preenchimento da ficha e incompletude dos dados da ficha de notificação. Os dados foram obtidos diretamente da Secretaria de Saúde do estado para garantir a melhor qualidade possível.

Tendo em vista os resultados deste estudo, torna-se evidente a necessidade de atualização e efetiva implementação de protocolos institucionais alinhados com as melhores evidências científicas nos serviços de saúde. Neste contexto, a capacitação dos profissionais pré-natalistas no manejo e notificação dos casos de sífilis durante a gestação é estritamente necessária. Ressalta-se que estas são ações imprescindíveis rumo à meta global de redução da sífilis congênita.

## CONCLUSÕES

Os achados desta pesquisa demonstram elevadas proporções de diagnóstico e tratamento inadequados de sífilis ao longo da gestação. Entretanto, houve aumento no número de notificações de sífilis latente em gestantes e no tratamento com penicilina benzatina na dose de 7.200.000 UI, o que sugere melhora da afinidade entre a prática profissional e as recomendações mais atuais. Também foi observado aumento na completitude dos dados da ficha de notificação nas variáveis de classificação clínica e tratamento, sugerindo melhora no processo de notificação ao longo dos anos.
